# The Accuracy of New and Aged Mechanical Torque Devices Employed in Five Dental Implant Systems

**DOI:** 10.1155/2017/8652720

**Published:** 2017-11-07

**Authors:** Mehmet Ali Erdem, Burcin Karatasli, Onur Dinçer Kose, Taha Emre Kose, Erhan Çene, Serhan Aydın Aya, Abdulkadir Burak Cankaya

**Affiliations:** ^1^Department of Oral and Maxillofacial Surgery, Faculty of Dentistry, Istanbul University, Istanbul, Turkey; ^2^Department of Prosthodontics, Faculty of Dentistry, Istanbul University, Istanbul, Turkey; ^3^Sultangazi Oral and Mouth Health Care Center, Istanbul, Turkey; ^4^Department of Dentomaxillofacial Radiology, Faculty of Dentistry, Recep Tayyip Erdogan University, Rize, Turkey; ^5^Department of Statistics, Yıldız Technical University, Istanbul, Turkey; ^6^Department of Mechanical Engineering, Faculty of Mechanical Engineering, Istanbul Technical University, Istanbul, Turkey

## Abstract

**Purpose:**

Friction-style and spring-style torque wrenches are used to tighten implant abutments and prosthetic screws. The mechanical stability of these torque wrenches is crucial for the implant–abutment connection. The purposes of this study were to assess the performance of five brands (Straumann, Zimmer, Implant KA, Bredent, and Biohorizons) of wrench and to evaluate possible changes in applied torque values of aged wrenches.

**Materials and Methods:**

Five new and aged wrenches that had been used approximately 250 times in a 1-year period were tested. The torque applied by friction- and spring-style wrenches was measured with a specially designed strain gauge indicator. Descriptive statistics, the one-sample* t*-test, and the independent-samples* t*-test were used to analyze values obtained from all torque wrenches.

**Results:**

The accuracy of new and aged torque devices of all brands except Bredent differed significantly from the target values, but the mean values for aged and new wrenches did not differ significantly from each other (*p* > 0.05). Values for the spring- and friction-type torque wrenches deviated from the target values by 11.6% and 10.2%, respectively.

**Conclusion:**

The accuracy of aged torque wrenches is adequate for prosthetic screw tightening, but that of new torque wrenches is unsatisfactory and must be examined carefully before delivery.

## 1. Introduction

The success of dental implant therapy may be evaluated in many different ways, including the effectiveness of immobilization, the acceptability of radiographic images, the acceptability of the extent of vertical bone loss, and the absence of surgical and prosthetic complications [1]. Prosthetic procedures commence after successful osseointegration, and many biological and mechanical factors affect the long-term clinical outcomes. Mechanical success is associated directly with continuity of the implant–abutment connection. The most important factors influencing such continuity are the preload applied, the accuracy of implant component integration, and the absence of rotation at the implant–abutment interface [2].

Torque devices are screw-bearing systems that deliver the force necessary to ensure connection of the implant–abutment complex. Torque wrenches are used to preload the abutment screws holding implant components together. Strain preload commences when a torque device is used to initially tighten an abutment screw. Strain on the screw forces the implant and abutment together, allowing the screw to resist external shear loads and fatigue [3]. Creation of an adequate compressive force is the principal manner by which abutment screws are preloaded [4]. Tight stable connections are important to ensure functional continuity [5, 6]. The required preload depends on various factors, including the applied torque, the alloy of which the screw is made, screw head design, the finish of the abutment surface, and the lubricant employed [7, 8].

The clamping force affects the mechanical properties of the screw joint [4]. The application of inadequate force may allow the screw to loosen under functional loads. On the other hand, forces that are too great may trigger screw fracture or flattening of the screw threads [4, 5]. Therefore, the force applied to the abutment screw must be greater than that required for separation [4]. The torque that can be applied is limited by screw durability, the amount of torque that can be delivered at the required speed (mechanical limitations), and the strain that the bone–implant interface can bear (the biological limit) [3]. The torque applied must be that recommended by the manufacturer. The torque applied to an abutment screw by a torque wrench should be within 10% of the target value. Biohorizons Inc. stated that the optimal torque value should be within 5% of the target value. Institute Straumann AG reported that their torque wrench was adequate, with torque values within 2 N·cm of the target values [9]. Shafi and Mohamed [3] found that torque devices affected the long-term performance of implant-supported prostheses and that the microgap decreased as the applied torque increased.

If the screw joint is inadequate, strain, bending, and compression forces can loosen the screw and the prosthesis [3]. Successful screw joint construction requires consideration not only of mechanical factors, but also of the status of soft tissues around the implant site and the possibility of alveolar bone loss. Insufficient preloading and inadequate screw tightening create stress and compromise stability [3]. In other words, any mismatch between the implant and the abutment delivers forces to peri-implant bony tissue, which can cause locking fractures, locking of the abutment and screw, loosening of the screw and prosthesis, bone microfractures, partial ischemia, loss of crestal bone, peri-implant mucositis, peri-implantitis, and even loss of osseointegration [3, 10].

Although one can find many studies of the effect of autoclaving on the accuracy of torque devices, few studies have examined the effects of time-dependent factors.

The aim of the present study was to explore the accuracies of new and aged torque wrenches and to compare the amounts of torque delivered by new devices and devices exposed to aging procedures.

## 2. Materials and Methods

**Figure 1:**
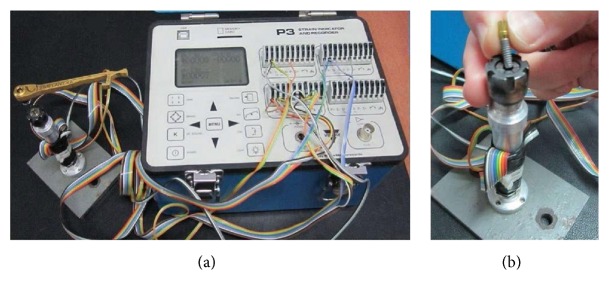
The P3 Strain Indicator and Recorder (a), and positioning of the implant in the device (b).

We evaluated five mechanical torque-application devices: the Straumann (Straumann Institute, Waldenburg, Switzerland), the Zimmer (Zimmer Dental, Carlsbad, CA, USA), the Implant KA (Mode Medical, Istanbul, Turkey), the Bredent (KG GmbH & Co., Senden, Germany), and the Biohorizons (Maestro Dental Implants, Birmingham, AL, USA). We examined five new and five used (250 times over 1 year) devices of each type (thus, 25 devices in total). Ten devices (Straumann, Implant KA) were spring-type and 15 (Biohorizons, Bredent, Zimmer) were friction-type. All devices have handles and holes for the insertion of screwdrivers produced by the manufacturers. We used one implant body, one abutment, and one screwdriver when studying each device. We used new screws for each measurement. We employed a total-bridge strain gauge (P3 Strain Indicator and Recorder; VPG, Micro-Measurements, Wendell, NC, USA) (Figure 1) to measure the torque applied by each device. A four-channel strain measuring device was used to measure torque values. Only three channels were used actively; one was used to measure the mean torque and the other two were used to measure mesiodistal and buccolingual tipping strain. The strain gauges were placed vertically to measure lateral forces and horizontally to measure torsional forces. Lateral forces were measured to eliminate any tipping, thereby ensuring that torque was applied only in the vertical direction.

When strain gauges are attached to equipment to determine stress levels and distributions, the ideal calibration method is to load the equipment at a known stress level and distribution, and to monitor the output of the installed strain gauges. Strain gauge–based transducers were calibrated by applying the appropriate dead-weight load to the torque arm or by using a torque wrench calibration machine and measuring the output. The first step of the calibration process was to amplify and filter the output of the bridge, while using the potentiometer of the amplifier to adjust and balance the output. A known force was then applied to the tube in all three directions, and the output values were recorded.

The implant body, abutment, and screw were attached to the strain gauge, which was set to zero before each test. All measurements were repeated five times; each device was tested in a separate session. All forces were applied by a single experienced operator to the limits recommended by the manufacturers. The operator was blinded to values measured by the P3 strain gauge.

The torque levels recommended by the manufacturers were 35 N·cm for the Straumann device; 30 N·cm for the Zimmer, Biohorizons, and Bredent devices; and 25 N·cm for the Implant KA device. The maximum torques applied with each device were recorded (in N·cm) by a clinician with reference to the strain gauge. We recorded the preload applied to the abutment screw, and the extents of right-to-left and front-to-back tilting. We measured only the preload values applied to the abutment screws. The mean torques delivered by each device were compared in terms of new/used status and accuracy. Descriptive statistics, the one-sample* t*-test, and the independent-samples* t*-test were used to compare the results; the alpha value was set to 0.05. IBM SPSS 21 software was used for statistical analysis.

## 3. Results

Table 1 shows the target, minimum, maximum, and mean values for all new and aged wrenches. The differences between new and aged wrenches were assessed using independent-samples* t*-test. Although slight differences were apparent between aged and new wrenches, statistical significance was not attained (Table 1).

For all wrenches except new Bredent, the mean values were less than the target values.  Table 2 shows the accuracy of aged and new wrenches. The one-sample *t*-test was used to determine whether the wrenches achieved the target values. Except for the Bredent, all aged and new wrenches applied torques that differed significantly from the target values.

Table 3 shows the differences between the mean and target values in N·cm and as percentages.

**Figure 2:**
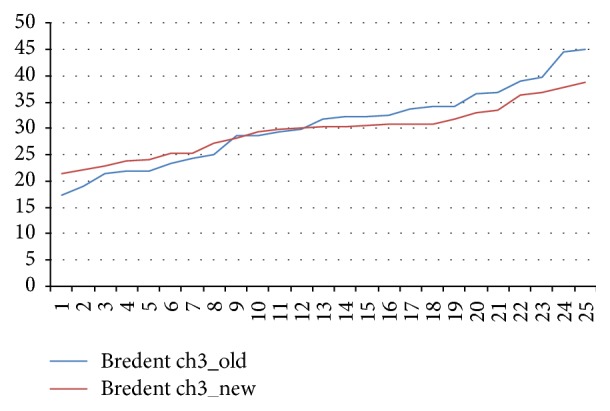
Torques delivered by aged and new Bredent ratchet wrenches.

The mean values for new and aged Bredent wrenches differed by only 1% from the target value which is not statistically different (*p* > 0.05; Figure 2).

**Figure 3:**
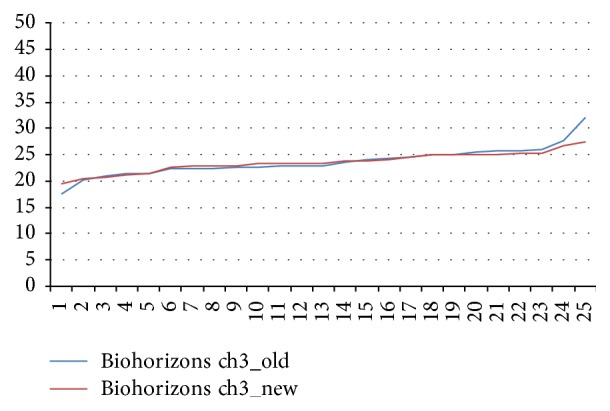
Torques delivered by aged and new Biohorizons ratchet wrenches.

The mean values for the new and aged Biohorizons wrenches differed by 21% from the target value (*p* < 0.05; Figure 3).

**Figure 4:**
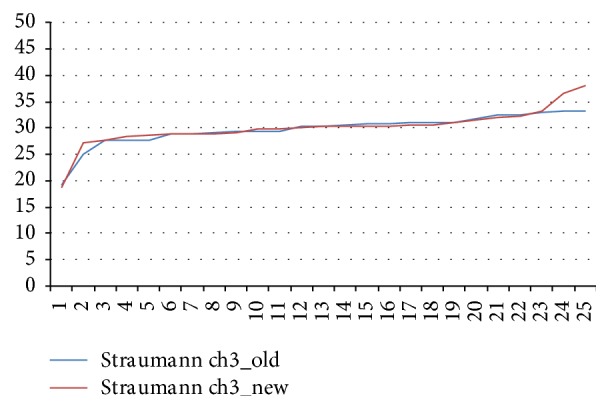
Torques delivered by aged and new Straumann ratchet wrenches.

The mean values for the new and aged Straumann wrenches differed by 14.34% from the target value (*p* < 0.05; Figure 4).

**Figure 5:**
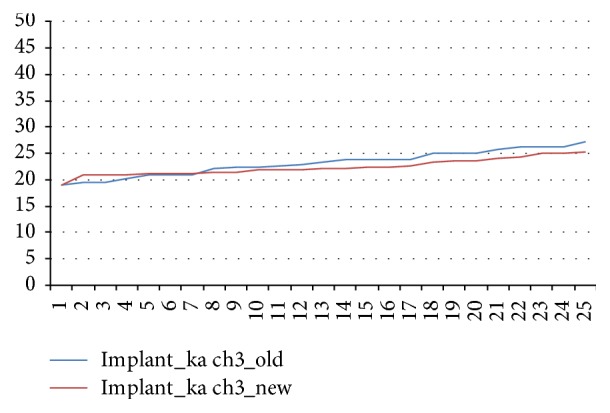
Torques delivered by aged and new Implant KA ratchet wrenches.

The mean values for the new and aged Implant KA wrenches differed by 8.9% from the target value (*p* < 0.05; Figure 5).

**Figure 6:**
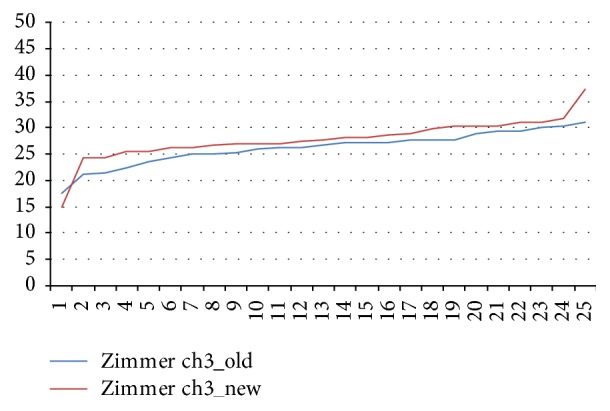
Torques delivered by aged and new Zimmer ratchet wrenches.

The mean values for new and aged Zimmer wrenches differed by 9.89% from the target value (*p* < 0.05; Figure 6).

The spring- and friction-type torque wrenches deviated from the target values by 11.6% and 10.2%, respectively.

## 4. Discussion

Application of the optimum torsional moment to the implant–abutment complex is critical for long-term successful prosthetic implant restoration. The implant–abutment connection loosens over time, resulting in microgaps, bacterial colonization, and peri-implantitis. Over time, microgaps progress to macrogaps. In this situation, the surface connection between the implant and abutment is lost, leading to the exertion of abnormally directed forces to the screw. These phenomena cause complications, such as inflammation/infection of the soft tissues and fracture of the screw [11]. As hand-held drivers do not adequately tighten abutments, the use of mechanical torque-limiting devices has become standard [12]. Application of the optimum torsional force to the implant–abutment connection, ideally using a torque-calibrated ratchet wrench, is crucial [11].

Two types of mechanical torque device are used in implant dentistry: friction- and spring-type devices [13]. The former are also termed toggle-type devices and the latter are also termed beam-type devices [12]. Friction-type devices are hexagonal wrenches with handles that release the applied force when the target torque value is attained. Spring-type devices have scales, and the clinician stops the application of force when the target torque is attained [13].

Strain gauges that measure electrical resistance yield accurate, stable, reliable, and reproducible data [3, 14]. Such gauges measure strain in different directions. Torsional stress can be measured by connecting four strain gauges to a full-bridge strain indicator. A four-channel strain measuring device was used to measure torque values in this study.

Spring-type wrenches are more accurate than friction-type devices [15]. Spring-type devices can be used to apply various torques, whereas friction-type devices deliver only the single torque set by the manufacturers [15]. In addition, spring-type wrenches can be used to place various types of implant [16]. Repetitive clinical use and repeated sterilization cycles affect the torques delivered by mechanical wrenches [12]. Friction-type devices may become corroded over time, rendering the applied torques inappropriate [17]. Autoclaving congeals the lubricants present in friction-type devices, causing the applied torque to increase [18].

Spring-type wrenches are not greatly affected by sterilization [12]. In the present study, we examined both types of device and found no significant difference between new and aged torque wrenches of any brand. Spring- and friction-type torque wrenches showed similar deviations from the target torque values (11.6% and 10.2%, resp.).

The torques applied by used and new wrenches differed from the target values, with the exception of the Bredent device. The most accurate new mechanical torque device was the Bredent (1.18% variation), followed by the Zimmer (7.19%), Implant KA (10.44%), Straumann (13.91%), and Biohorizons (21.51%) devices. The most accurate used wrench was the Bredent (1.79%), followed by the Implant KA (7.36%), Zimmer (12.59%), Straumann (14.78%), and Biohorizons (20.98%) wrenches. Biohorizons wrenches exhibited the greatest variation, deviating by almost 21% from the target value. Bredent wrenches (used and new) were the most consistent, deviating by only ca. 1% from the target value. We found no significant difference in torque output between used and new devices. (*p* > 0.05; Table 1). Yilmaz et al. [9] reported that Biohorizons, Zimmer, and Straumann torque wrenches subjected to 100 autoclaving cycles showed no significant difference in applied torque values, and that the torque applied by new and aged torque wrenches was within 10% of the target values.

The Straumann and Implant KA hand-held mechanical torque devices have spring activated sleeves and calibrated scales. Torque is applied until the required force is obtained. The devices have no mechanical stop; thus, the torque that can be applied is not limited. Çehreli et al. [4] tested 15 Straumann mechanical torque devices divided into three subgroups: new, used 50–200 times, and used 500–1,000 times. A slight decrease in applied torque was evident after 500–1,000 uses. Deformation was always within the elastic limit, suggesting that screw loosening may not be of concern when such devices are employed. Çehreli et al. [4] suggested that the observed decrease in torque was attributable to fatigue in the region where the ratchet is connected to the spring. We found that the mean torque delivered by used Straumann devices was slightly lower than that of new devices, but the difference was not significant (*p* = 0.736). In addition, the mean torque delivered by used and new Implant KA wrenches did not differ significantly (*p* = 0.177; Table 1).

Standlee et al. [19] found that two of three wrenches exerted torques within 10% of the target values. We found that the torques exerted by the Implant KA and Straumann spring-type devices varied by up to 8% and 14%, respectively. The torque output of friction-type devices (Bredent, Biohorizons, and Zimmer) varied by up to 0.3%, 21%, and 9%, respectively. Vallee et al. [15] found that the torque output values of friction-style devices were below specification, and those of spring-style devices were slightly higher than specification. McCracken et al. [12] compared friction- and spring-type wrenches and found that the mean torques delivered did not differ significantly, but that the torques produced by friction-type wrenches varied more than did those produced by spring-type wrenches. With the exception of the Bredent wrench, we found that the mean torques exerted by friction- and spring-type devices were below specification.

We evaluated the accuracy of various brands of mechanical torque-limiting device, which is affected by clinical use. However, an important limitation of our study is that we had no information on the extent of prior device utilization or the numbers of sterilization cycles to which the wrenches had been subjected. These data were given by the manufacturers.

## 5. Conclusion

Except for one brand, no torque device tested achieved the exact manufacturer-recommended value. The accuracies of new and aged torque wrenches were equivalent, indicating that these wrenches are not easily affected by aging procedures.

We found no significant difference between new and aged devices of any brand. However, and more importantly, the accuracy of the new devices was unsatisfactory and must be carefully examined before delivery.

## Figures and Tables

**Table 1:** Minimum, maximum, and mean values for all new and aged wrenches.

Implant system	Minimum value	Maximum value	Mean value	Std. deviation	*p* value
Biohorizons (aged)	17.570	32.024	23.703	2.770	*p* > 0.05 (0.814)
Biohorizons (new)	19.554	27.489	23.544	1.888	

Bredent (aged)	17.287	45.060	30.39	1.5069	*p* > 0.05 (0.619)
Bredent (new)	21.538	38.825	29.643	0.9666	

Implant KA (aged)	18.9878	27.2064	23.1594	2.3690	*p* > 0.05 (0.177)
Implant KA (new)	18.9878	25.2226	22.3886	1.5173	

Straumann (aged)	19.27	33.16	29.8250	2.9557	*p* > 0.05 (0.736)
Straumann (new)	18.70	37.98	30.1311	3.4132	

Zimmer (aged)	17.5708	31.1740	26.2201	3.2001	*p* > 0.05 (0.113)
Zimmer (new)	15.0202	37.4088	27.8412	3.8774	

**Table 2:** Accuracies of new and aged wrenches.

Implant system	Target value	*t*	95% confidence interval of the difference		Mean difference	*p* value
			Lower	Upper		
Biohorizons (used)	30	−11.363	−7.4400	−5.1527	−6.29642	*p* < 0.05
Biohorizons (new)	30	−17.090	−7.2347	−5.6755	−6.4551	*p* < 0.05

Bredent (used)	30	0.358	−2.5709	3.6493	0.5391	*p* < 0.05
Bredent (new)	30	−0.369	−2.3513	1.6386	−0.3563	*p* < 0.05

Implant KA (used)	25	−3.885	−2.8184	−0.8626	−1.8405	*p* < 0.05
Implant KA (new)	25	−8.605	−3.2377	−1.985	−2.6114	*p* < 0.05

Straumann (used)	35	−8.754	−6.3950	−3.9549	−5.1749	*p* < 0.05
Straumann (new)	35	−7.132	−6.2778	−3.4599	−4.8689	*p* < 0.05

Zimmer (used)	30	−5.906	−5.1007	−2.4588	−3.7798	*p* < 0.05
Zimmer (new)	30	−2.784	−3.7592	−0.5582	−2.1587	*p* < 0.05

**Table 3:** Differences between mean values and target values in N·cm and as percentages.

	Biohorizons	Bredent	Implant KA	Straumann	Zimmer
Target value (N·cm)	30	30	25	35	30

All wrenches					
Mean value	23.624	30.091	22.774	29.978	27.031
Difference	−6.376	0.091	−2.226	−5.022	−2.969
Difference%	−21.253	0.305	−8.904	−14.348	−9.898

Aged wrenches					
Mean value	23.704	30.539	23.159	29.825	26.220
Difference	−6.296	0.539	−1.841	−5.175	−3.780
Difference%	−20.988	1.797	−7.362	−14.786	−12.599

New wrenches					
Mean value	23.545	29.644	22.389	30.131	27.841
Difference	−6.455	−0.356	−2.611	−4.869	−2.159
Difference%	−21.517	−1.188	−10.446	−13.911	−7.196
